# *EPAS1* variants in high altitude Tibetan wolves were selectively introgressed into highland dogs

**DOI:** 10.7717/peerj.3522

**Published:** 2017-07-12

**Authors:** Bridgett vonHoldt, Zhenxin Fan, Diego Ortega-Del Vecchyo, Robert K. Wayne

**Affiliations:** 1 Ecology & Evolutionary Biology, Princeton University, Princeton, NJ, United States of America; 2 College of Life Sciences, Sichuan University, Chengdu, China; 3 Department of Integrative Biology, University of California, Berkeley, CA, United States of America; 4 Ecology & Evolutionary Biology, University of California, Los Angeles, CA, United States of America

**Keywords:** Adaptive introgression, Elevation, Domestic dogs

## Abstract

**Background:**

Admixture can facilitate adaptation. For example, black wolves have obtained the variant causing black coat color through past hybridization with domestic dogs and have higher fitness than gray colored wolves. Another recent example of the transfer of adaptive variation between the two species has been suggested by the similarity between high altitude Tibetan mastiffs and wolves at the *EPAS1* gene, a transcription factor induced in low oxygen environments.

**Methods:**

Here, we investigate the directionality of admixture in *EPAS1* between 28 reference highland gray wolves, 15 reference domestic dogs, and 21 putatively admixed highland wolves. This experimental design represents an expanded sample of Asian dogs and wolves from previous studies. Admixture was inferred using 17,709 publicly available SNP genotypes on canine chromosome 10. We additionally conducted a scan for positive selection in the highland dog genome.

**Results:**

We find an excess of highland gray wolf ancestry at the *EPAS1* locus in highland domestic dogs, suggesting adaptive introgression from wolves to dogs. The signal of admixture is limited in genomic extent to a small region on chromosome 10, indicating that it is the focus of selection in an oxygen-limited environment.

**Discussion:**

Our results suggest that an adaptive variant of *EPAS1* in highland wolves was transferred to highland dogs, carrying linked variants that potentially function in hypoxia response at high elevation. The intertwined history of dogs and wolves ensures a unique evolutionary dynamic where variants that have appeared in the history of either species can be tested for their effects on fitness under natural and artificial selection. Such coupled evolutionary histories may be key to the persistence of wild canines and their domesticated kin given the increasing anthropogenic modifications that characterize the future of both species.

## Introduction

A novel mechanism that can promote accelerated adaptation is the spread of beneficial alleles through admixture between closely related species. Admixed individuals often display novel gene combinations that can be tested by natural selection in a distinct ecological context (Arnold, 2016) or facilitate expansion into new ecological niches enhancing evolutionary persistence (Becker et al., 2013; Hedrick, 2013). Though adaptation may occur more quickly from standing variation, admixture is the only process that can transfer entire co-adapted multi-gene complexes (Hedrick, 2013). Examples of adaptive introgression include rodenticide warfarin resistance in mice (Song et al., 2011), black coat color in wolves (Anderson et al., 2009), beak morphology in Darwin’s finches (Grant & Grant, 2010), immunity genes in humans (Mendez, Watkins & Hammer, 2012a; Mendez, Watkins & Hammer, 2012b; Abi-Rached et al., 2011) and collar color in manikins (MacDonald et l., 2001).

Recently, the genetic basis underlying adaptation to high altitude in Tibetans was elucidated. Although several genes seem to be involved in the response, *EPAS1* showed the strongest signal of selection in Tibetans (Beall et al., 2010; Simonson et al., 2010; Peng et al., 2011; Xu et al., 2011), and was obtained through adaptive introgression from Denosovian-like ancestors (Huerta-Sánchez et al., 2014). *EPAS1* (or HIF2α) is a transcription factor induced in oxygen-limited (hypoxic) environments which dampens the erythropoietic response and is under natural selection (Beall et al., 2010). Previously, we showed *EPAS1* experienced strong positive selection in highland Chinese wolves as indicated by divergent allele frequencies at non-synonymous SNPs (Zhang et al., 2014). Likewise, a similar signal on *EPAS1* in the Tibetan mastiff and other highland dog breeds was detected (Gou et al., 2014). Recently, Miao, Wang & Li (2016) identified that the Tibetan mastiff *EPAS1* locus was adaptively introgressed using the *D* statistic (Green et al., 2010; Durand et al., 2011). However, inferring the *D* statistic is challenging for small genomic regions as it shows more extreme values if the effective population size is low and if the interrogated regions have low genetic diversity (Martin, Davey & Jiggins, 2015), such as those found in high altitude wolves (Fan et al., 2016). Further, a single *D*-statistic test cannot be used to directly infer the direction of the introgression event and the authors suggest it likely was transferred from wolves to dogs since wolves had a more ancient history on the Tibetan Plateau. Finally, this survey was limited to Tibetan mastiffs as examples of high altitude dogs and only included a single low elevation dog breed for comparison.

**Figure 1:**
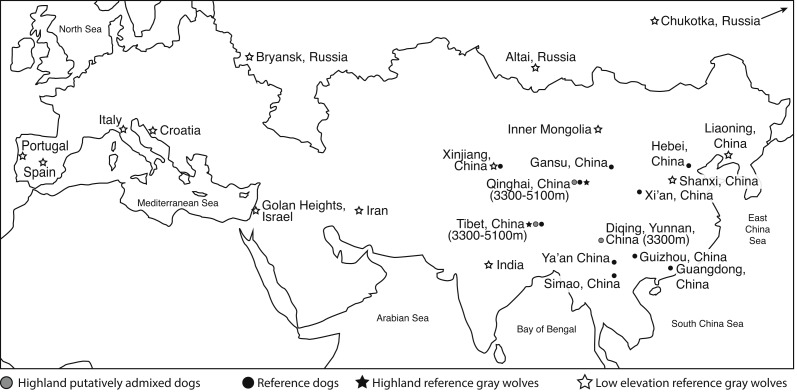
Eurasian map of the general geographic location from which genome samples were derived. Elevation is provided. Two reference dogs (Dingo and Basenji) are not included in this map. See Table S1 for more details.

Here, we investigate the hypothesis that *EPAS1* in domestic highland dogs was obtained through introgression from highland wolves using a larger panel of reference canids. As such, we focused on the chromosome where this candidate gene resides, canine chromosome 10. We use complete sequence data of chromosome 10 to specifically assess if dogs and wolves derived *EPAS1* mutations through introgression, and to define which of the two species was the donor if there was an introgression event. Dogs have dispersed into new environments along with the expansion of human civilization. According to previous studies of the history of human settlement on the Tibetan Plateau, the initial colonization occurred during the Paleolithic era (Zhao et al., 2009; Qi et al., 2013; Lu et al., 2016). Therefore, given the recent arrival of dogs to the Tibetan Plateau, the transfer of hypoxia adaptations from dogs to wolves would be a striking second example of variants selected under domestication transforming a related wild population. The first example is the *K* locus (a canine beta-defensin gene), which is a mutation that causes to canine melanism that originated in the dog genome and was transferred through introgression to North American gray wolves over 10,000 years ago (Anderson et al., 2009). Alternatively, if the mutations originated in wolves, which have a much longer history in the Tibetan Plateau (Brantingham, Rhode & Madsen, 2010; Chen et al., 2015), then this would be the first example of adaptive introgression from wolves to dogs. A third possibility is the independent evolution of high elevation adaptations in dogs and wolves, a result which would highlight the relative mutability of *EPAS1* as a response to environmental challenges. To better distinguish among these hypotheses, we use an expanded representation of dogs and wolves from both high and low elevation populations, and an analytical method that specifically assigns genomic regions to either species. We conclude that the recent arrival of dogs and the high diversity of and the deep divergence among *EPAS1* haplotypes in highland wolves suggest the high altitude adapted variant originated in the highland wolves and was transferred to dogs, rather than evolving independently in both highland dogs and wolves.

## Materials & Methods

### Samples and genome sequence data

To determine if highland dogs inherited a beneficial allele through admixture with wolves, we obtained publically available genome sequence and SNP genotype data from 21 highland (>3,300 m) dogs (*n* = 10 indigenous dogs from a putatively admixed population, *n* = 11 Tibetan mastiffs) in China to compare to 15 reference dogs (*n* = 13 indigenous Chinese dogs; *n* = 1 basenji, *n* = 1 dingo), 22 low elevation Old World (OW) wolves, and six highland wolves from China (Fig. 1, Table S1). For this study, we obtained the genomic sequence and SNP variants for two genomes: one low elevation wolf from Inner Mongolia (im5) and one highland wolf from Tibet (ti3) (Table S1). The whole genome sequencing was performed using an Illumina Hiseq 2,000 at Novogene (China). Two paired-end libraries with insert sizes of 300∼500 bp were generated for each sample. Library preparation and sequencing was performed according to manufacturer’s protocols. Our newly generated sequence data can be found on NCBI’s Sequence Read Archive (SRA) under the accession number SRP096612. The 150 bp pair-end reads of each sample were aligned to the dog genome (CanFam3.1) using Bowtie 2 (Langmead & Salzberg, 2012) under the local alignment algorithm with the very sensitive model. Default options were used for all other parameters. Then, we applied Picard and GATK toolsets (DePristo et al., 2011) to process the alignments to identify SNPs. The pipeline is the same as used in our previous studies (Freedman et al., 2014; Zhang et al., 2014; Fan et al., 2016).

To obtain a set of polymorphic SNP genotypes, we excluded monomorphic sites and applied a strict inclusion threshold of no missing data to obtain 17,709 SNPs on canine chromosome 10. Further, to increase SNP density around the candidate gene *EPAS1*, we allowed on average 14% missing data, which provided an additional 1,113 SNP genotypes (total number of SNPs = 18,792). To assess if our assumed reference dogs were indeed non-admixed, we used the *Ancestry* function in Galaxy’s Genomic Diversity toolkit for *K* = 2–4 (Alexander, Novembre & Lange, 2009; Afgan et al., 2016) and performed a complete population structure analysis for all samples with 17,709 SNP genotypes.

### Admixed ancestry analysis

To better explore the hypothesized introgression of *EPAS1* in to highland dogs, we utilized ancestry information from a larger reference panel of dogs and gray wolves, and used a different methodological approach than previous work. Specifically, following methods in vonHoldt et al. (2011), we estimated the degree of per-SNP genetic differentiation (F_ST_) between the reference dog and Old World wolves and employed a 2 standard deviation (SD) threshold to identify SNPs with significantly high levels of divergence. We then identified SNPs with strongly divergent allele frequencies between the two reference groups of dogs and wolves. Namely, sites with the highest F_ST_ values, as defined by the 2SD threshold, were classified as ancestry informative markers (AIMs).

We assigned either dog or wolf ancestry to chromosome 10 SNPs from 21 highland dogs that are putatively admixed with OW wolves. Samples were assessed for outlier patterns using principal component analysis (PCA) in Galaxy’s Genomic Diversity toolkit (Afgan et al., 2016) on both the set of 17,709 and 980 AIM SNPs. Further, we surveyed local ancestry proportions for highland dogs using the program *Admixture* in Galaxy’s Genomic Diversity toolkit from the estimated genotype, two reference populations of dogs and wolves, and a switch penalty value of 10 to estimate per-site ancestry of the putatively admixed highland dogs (Table S1). We then surveyed average wolf ancestry across chromosome 10 in 100-SNP windows with a 50-SNP step in the putatively admixed highland dogs. Outlier regions were identified by a simple outlier threshold of wolf ancestry proportion >2SD above the chromosomal level (average wolf proportion, SD = 0.032, 0.09). We further repeated the PCA and completed a population structure analysis by using the *Ancestry* function in Galaxy (Alexander, Novembre & Lange, 2009) for SNP genotypes within any putatively introgressed regions. To phase our SNP genotypes using *SHAPEIT* (Delaneau, Marchini & Zagury, 2012), we constructed a genetic map file as the ratio of inter-SNP distances to the position of the last SNP queried on chromosome 10. Additionally, we phased SNP genotypes using the program *PCAdmix* and plotted the per-individual loadings for the first two coordinates (Henn et al., 2012). Lastly, we applied a haplotype-based test (Mendez, Watkins & Hammer, 2012a; Mendez, Watkins & Hammer, 2012b) to determine if the shared *EPAS1* haplotype in highland dogs and wolves was due to an adaptive introgression event or the alternative hypothesis that selection acted on a haplotype present in the ancestral population. We utilized a recently inferred genetic map (Campbell et al., 2016). We assume a generation time of three years (Freedman et al., 2014; Fan et al., 2016) and that all recombination events are detectable.

Variant effect predictions were conducted with transcripts annotated from the reference dog genome (CanFam 3.1), with SIFT (*Sorting Intolerant From Tolerant*) scores on non-synonymous substitutions, which range from 0 to 1, used to predict if the non-synonymous variants may be damaging. Specifically, if the SIFT score is ≤0.05 it is considered damaging, or tolerated when the score is >0.05 (McLaren et al., 2010).

### Scan for positive selection

We scanned the target genomic region for signals of positive selection in the highland dog genome compared to the reference dogs using cross population extended haplotype homozygosity (*XP-EHH*) tests (Sabeti et al., 2007). This analysis was based on the phased haplotypes from 836 SNPs that had no missing data within the putatively introgressed region containing *PRKCE* and *EPAS1*. Per-SNP F_ST_ was calculated as described above. We normalized both the F_ST_ and XP-EHH scores as *z*-scores with a mean of zero and standard deviation of 1. Their product represents a composite “bivariate percentile score”. We identified outlier loci as those in the 97.5th percentile or greater (*z*-score > 2) of the bivariate percentile score distribution.

**Figure 2:**
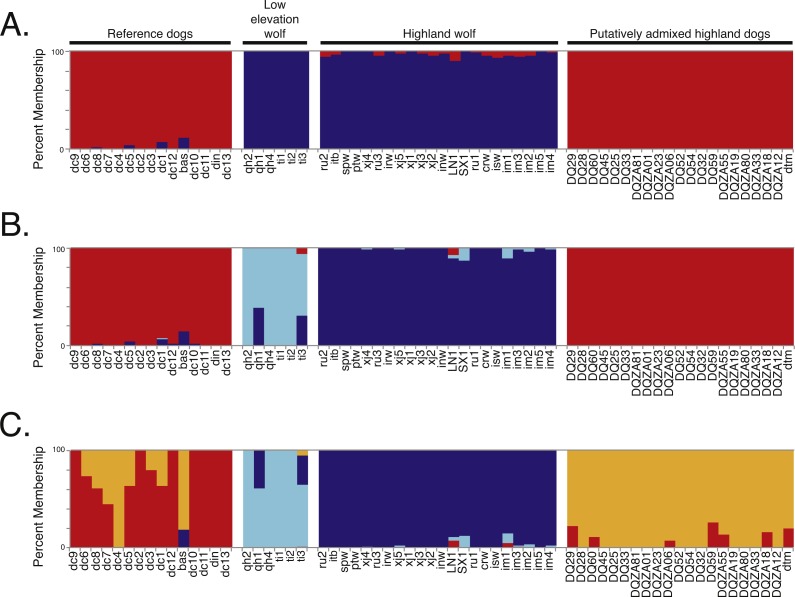
Genetic structure analysis of 64 canids for 17,709 SNPs across chromosome 10 for (A) *K* = 2, (B) *K* = 3, and (C) *K* = 4 assumed clusters. Black bars indicate distinct populations and sample IDs are provided along the *X*-axis.

**Figure 3:**
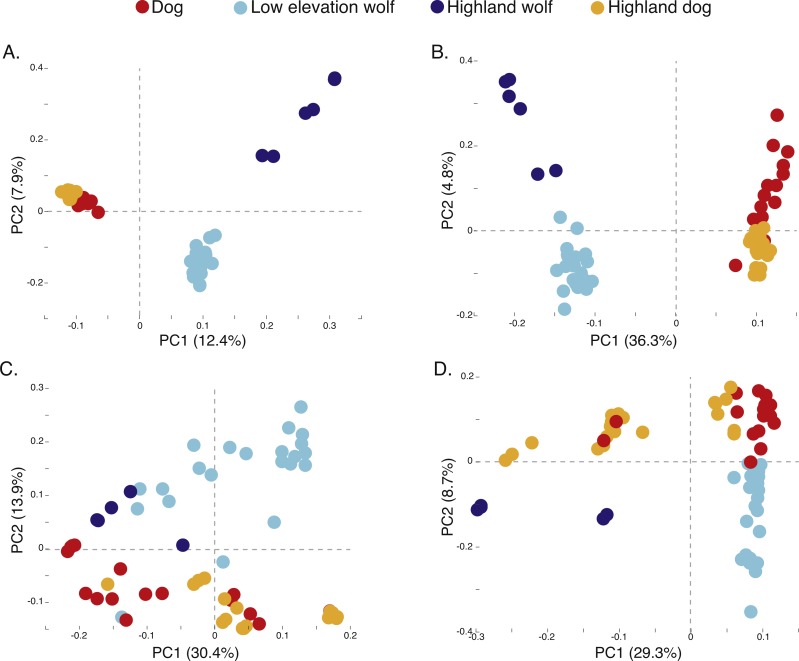
Principal component analysis of (A) 17,709 SNPs, (B) 980 AIMs, and across two ancestry outlier genes (C) MSRB3 (100 SNPs) and (D) *PRKCE/EPAS1* (167 SNPs).

## Results

### Two regions are enriched for wolf ancestry in highland dogs

To determine if admixture mapping is useful in searching for adaptively introgressed alleles in Chinese highland dogs, we first conducted a survey of genetic structure for 64 Old World canids along chromosome 10 (SNPs *n* = 17, 709) (Fig. 1; Table S1). We found that the reference wolves represent two genetic clusters, high and low elevation individuals, with little evidence for admixture with dogs (Figs. 2, 3A). We further identified the reference dogs to be largely non-admixed with wolves, with the only exception being the basenji (bas; Fig. 2). Although the highland and reference dogs were similar in the PCA analysis (Fig. 3A), these two populations were distinct at *K* = 4 and displayed varying degrees of admixture with each other (Fig. 2). Further, highland dogs contained low chromosome-wide proportions of wolf ancestry on chromosome 10 (max = 0.027) (Table 1).

**Figure 4:**
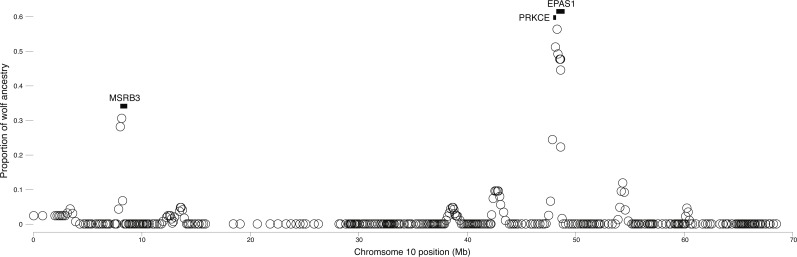
Local average wolf ancestry in a sliding window for highland dogs. Genes are indicated by black boxes.

**Figure 5:**
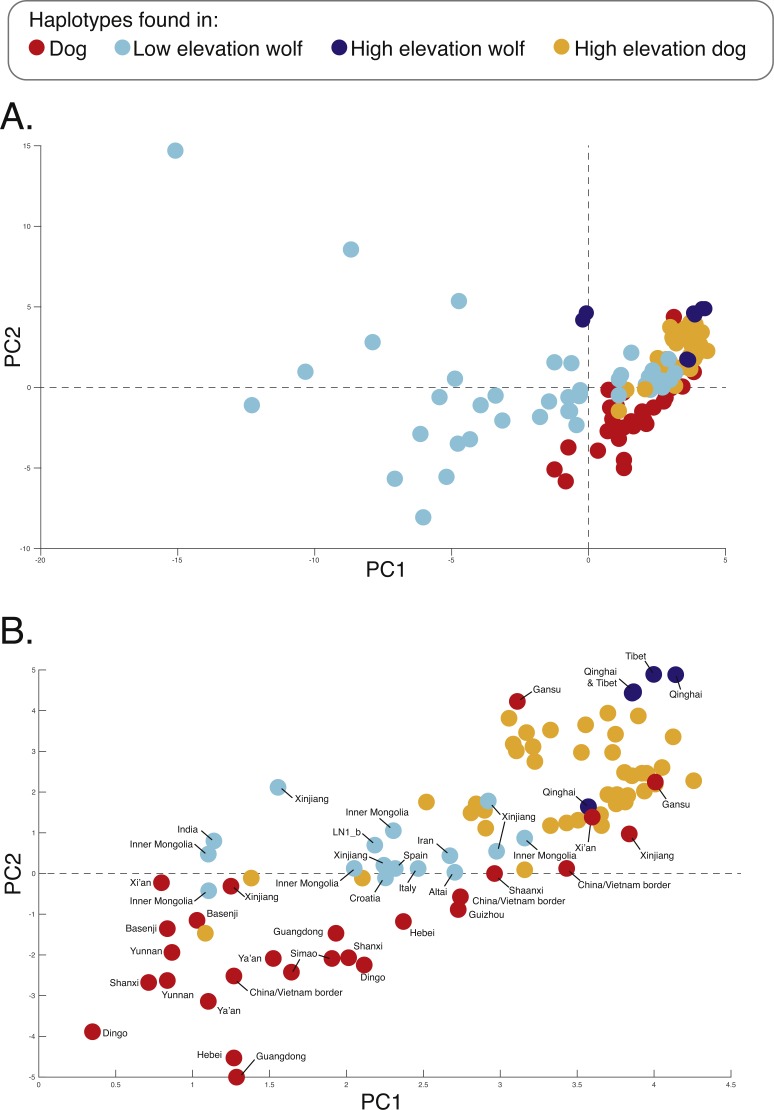
PCA of phased haplotypes for the putatively introgressed region containing the *PRKCE* and *EPAS1* genes for the (A) whole sample set and (B) those samples with PC1 > 0. Geographic origins of each haplotype are provided. Refer to Fig. 1 for the map and Table S1 for more sample details.

We surveyed ancestry across multiple dog and wolf populations based on 18,794 SNP genotypes on canine chromosome 10 to provide a higher density survey across the candidate gene, *EPAS1*. Using the 2SD threshold, we classified 980 F_ST_ outlier SNPs as AIMs that were informative for dog and wolf ancestry (all SNPs: F_ST_ average = 0.04, SD = 0.10, +2SD = 0.23; AIMs: F_ST_ average = 0.38, SD = 0.13), with an average density of 1 AIM per 70Kb across chromosome 10 (median = 26 Kb). Though non-model based, a PCA of 980 AIMs clearly identified four clusters, partitioned by species and elevation (Fig. 3B; Fig. S1). A sliding window analysis of AIMs along chromosome 10 revealed an enrichment of wolf ancestry in two regions (chr10: 8147438–8288850 containing the *MSRB3* gene, average wolf proportion = 0.278; chr10: 48151501–48761234: *PRKCE* and *EPAS1* genes, average wolf proportion = 0.355), thus identifying them as candidate outliers (Fig. 4). Genetic analysis of each outlier region varies in the degree of structure (Figs. 3C and 3D; Fig. S1) and observed outliers are found in three putatively introgressed genes. Following previous work and with the objective to explore the introgressed history of *EPAS1* in high elevation adaptation, we focused on the region containing *PRKCE* and *EPAS1* in subsequent analyses. We conducted a PCA of phased haplotypes from this genomic region that contains both an excess of wolf ancestry in dogs and two functionally-relevant candidate genes. Our objective was to identify the potential source population of this putatively introgressed region in highland dogs. Low elevation wolves are spatially discrete compared to those from the highland, where 10 of 12 haplotypes are nested within the putatively admixed highland dogs (Fig. 5). This result suggests that the putatively introgressed region in the highland dog genome originated from highland wolves in Qinghai and Tibet, China. From the phased haplotypes of the introgressed region containing *PRKCE* and *EPAS1*, we defined a 396 Kb subregion (n_SNPs_ = 467; chr10: 48305073–48700082) that had had a high proportion of wolf ancestry (wolf prop. > 0.45) across all highland dogs implying the origin of this region derives from admixture with highland gray wolves.

**Table 1:** Wolf ancestry proportions for the putatively admixed Old World highland dogs for 17,709 SNPs on chromosome 10. (Abbreviations: Prop., proportion)

Sample ID	Wolf prop.
DQ25	0.007
DQ28	0.017
DQ29	0.029
DQ32	0.002
DQ33	0.005
DQ45	0.010
DQ52	0.004
DQ54	0.002
DQ59	0.002
DQ60	0.008
DQZA01	0.015
DQZA06	0.021
DQZA12	0.006
DQZA18	0.006
DQZA19	0.041
DQZA23	0.011
DQZA33	0.012
DQZA55	0.002
DQZA80	0.006
DQZA81	0.006
dtm	0.016

To better understand functional variation in this introgressed region, we determined the presence of damaging variation. We restricted our assignment of functional changes to SNPs within this candidate subregion and found one missense damaging non-synonymous variants in each of the *PRKCE* and *EPAS1* genes (Table 2). The *PRKCE* variant was only observed in a single highland wolf (qh4), and the *EPAS1* variant found in a putatively admixed highland Tibetan mastiff (DQZA81) and one low elevation indigenous reference dog (dc5).

Using the recent canine genetic map, we inferred that the 60 Kb length block containing the *EPAS1* gene has a genetic map length of 0.032 cM. Consequently, the time until one of the two identical-by-descent copies of a *EPAS1* haplotype are broken by a recombination event is exponentially distributed with a parameter of (0.00032 *  2) per generation. We found that one of the two identical-by-descent copies of the *EPAS1* haplotype has a 50%, 95% and 99% probability of at least one recombination event in 3,261, 14,092 and 21,663 years, respectively. On the other hand, estimates of the divergence time of dogs and wolves range from 11,700 or 29,000 years ago depending on the mutation rate used (Freedman et al., 2014; Skoglund et al., 2015; Fan et al., 2016). These divergence time estimates imply that recombination would have broken one of the two identical by descent copies of the *EPAS1* haplotype at least once (with a 95% probability) if the ancestral haplotype was present in the population ancestral to dogs and wolves. Contrary to this expectation, highland dogs and wolves share the full 60 Kb *EPAS1* haplotype, which is evidence in favor of an adaptive introgression event rather than selection on an ancestral haplotype in common ancestor of dogs and wolves.

**Table 2:** Putative functional consequences of SNP variants within the introgressed subregion (chr10: 48305073–48700082) of admixed highland dogs that contains *PRKCE* and *EPAS1*.

CFA10 position	Allele	Gene	Consequence	Impact	Predicted SIFT phenotype (score)	Individuals carrying the allele
48,284,953	T	*PRKCE*	Missense	Moderate	Deleterious (0)	One highland wolf (qh4)
48,631,974	C	*EPAS1*	Missense	Moderate	Deleterious (0.02)	Tibetan mastiff (DQZA81), low elevation indigenous dog (dc5)

### Signal of selection in *EPAS1*

**Figure 6:**
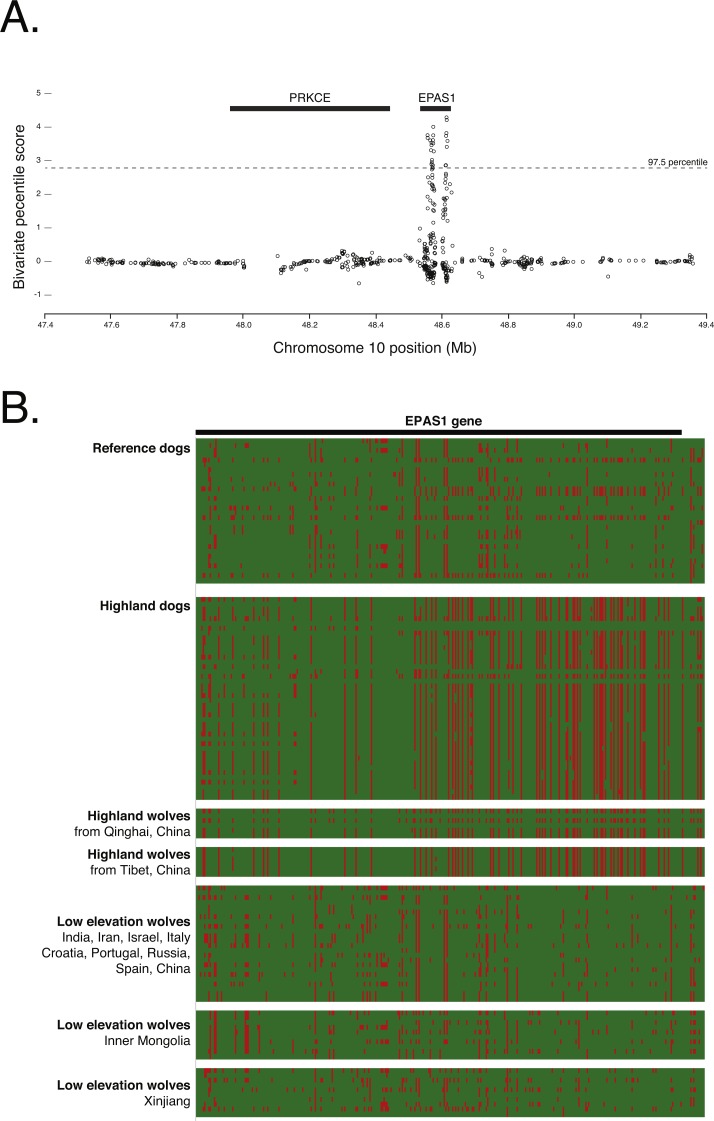
(A) Positive selection scan of 835 SNPs in the Old World highland dogs. (B) Phased haplotypes for 363 phased SNPs in *EPAS1* (as indicated by the black bar). Rows are individuals; columns are SNP positions. Red color indicates the canfam3.1 reference allele; green the alternate allele.

To confirm the signal of selection on *EPAS1* as detected in previous studies, we conducted a cross-population analysis of extended haplotype homozygosity and genetic differentiation between the highland and low elevation dog genomes. This analysis supports the previous finding that *EPAS1* is under strong positive selection (chr10: 48565127–48625694). However, this signal is discrete and does not encompass the adjacent gene, *PRKCE* (Fig. 6A), further supporting the specific role of *EPAS1* in high elevation adaptation in domestic dogs. Additionally, phasing the 363 SNPs in *EPAS1* also confirmed a shared haplotype between the highland dogs and wolves from Tibet (samples ti1, ti2, ti3) and Qinghai (samples qh1, qh2, qh4), China (Fig. 6B).

## Discussion

Adaptive introgression is an effective and rapid mechanism for species to adapt to new environments through the transmission of beneficial variants from related locally adapted taxa (Hedrick, 2013). Here, we highlight the role of adaptive introgression from Tibetan wolves to a diverse representation of high altitude dogs in China. We identified an introgressed haplotype containing the genes *EPAS1* and *PRKCE*. Though introgressed as part of the same haplotype, only *EPAS1* is under strong positive selection in highland dogs. However, the role of *PRKCE* in wild populations has yet to be explored. Experimentally, *PRKCE* null alleles in mice reduce pulmonary vasoconstriction in a hypoxic treatment (Littler et al., 2003). Analysis of diagnostic SNPs and the phased chromosomes revealed that the most likely source of the introgressed haplotype is from wolves in Tibet and Qinghai, China.

An analogous adaptive introgression process has been described between dogs and North American gray wolves. Specifically, the *K* locus, (a canine beta-defensin gene) has a dominantly inherited 3-bp mutation that causes black coat color in wolves and when heterozygous, confers higher fitness (Anderson et al., 2009; Coulson et al., 2011). This mutation, though Old World in origin, was transferred to North American gray wolves from New World dogs >10,000 years ago, yet no trace of dog ancestry remains in these wolves outside of the introgressed region (Anderson et al., 2009; Schweizer et al., 2016a; Schweizer et al., 2016b). Our finding of adaptive admixture in dogs, parallels the history of high altitude Tibetans whose *EPAS1* haplotype derives from admixture from Denisovans but whose genome is otherwise very similar to geographically proximate low altitude Han populations (average F_ST_ ∼ 0.02; Huerta-Sánchez et al., 2014). Given the absence of the *EPAS1* highland haplotype in the genome of dogs from lowland regions, parallel mutation in highland dogs and wolves followed by an independent selective sweep would be needed to explain our results. This scenario is less parsimonious than adaptive introgression. The length of the conserved highland haplotype in dogs and wolves is also consistent with adaptive introgression, instead of selection on a haplotype that has not been broken up by recombination since at least the divergence of dogs and wolves. Finally, both the high diversity of and divergence among *EPAS1* haplotypes suggest they evolved well before the arrival of dogs to the highlands about 10,000 years ago.

We identified two moderately deleterious missense variants in two genes in the candidate region containing *PRKCE* and *EPAS1*. One variant in *EPAS1* was restricted to the Tibetan mastiff, whereas the second variant was found in *PRKCE* in a single highland admixed dog and a single low elevation indigenous dog. *EPAS1*’s function is central to hypoxia adaptation, which can be mitigated through a physiological decrease in the oxygen demand, typically through an upregulation of ATP-producing pathways and reduced activity of ATP-demanding pathways (e.g., ion pumping and protein synthesis) (Storey & Storey, 1990; Guppy, Fuery & Flanigan, 1994; Gnaiger & Kuznetsov, 2002). Our results suggest that adaptive variation of *EPAS1* from highland wolves and variants in linked genes potentially facilitate the hypoxia response in highland dogs through adaptive introgression and that the *EPAS1* variant unique to Tibetan mastiffs is not likely the causative variant in highland dogs. Critically, we show that this adaptation in dogs did indeed derive through hybridization with highland wolves confirming that adaptive variation can be transferred in both directions from wolves to dogs and dogs to wolves. The intertwined history of dogs and wolves ensures a unique evolutionary dynamic where variants that have appeared in the history of either species can be tested for their effects on fitness under natural and artificial selection. Such coupled evolutionary histories may be key to the persistence of wild canines and their domesticated kin given the increasing anthropogenic modifications that characterize the past, present and future of both species.

##  Supplemental Information

10.7717/peerj.3522/supp-1 Supplemental Information 1 Supplemental figures and tables

10.7717/peerj.3522/supp-2 Supplemental Information 2 Structure membership Population membership proportions for 983 AIMs and for each ancestry outlier gene, MSRB3 and PRKCE/EPAS in high elevation dogs.

## Additional Information and Declarations

### Data Availability

The following information was supplied regarding data availability: The only new sequences collected for this manuscript can be found on SRA (NCBI, accession SRP096612).
