# Supplementary material for: EPAS1 variants in high altitude Tibetan wolves were selectively introgressed into highland dogs
Source: PeerJ. 2017 Jul 12;5:e3522. doi: 10.7717/peerj.3522 (PMC5510585; doi:10.7717/peerj.3522)
Supplement: Supplemental Information 1 [file peerj-05-3522-s001.docx]

**Supplemental Figures**

**Figure S1.** Genetic structure analysis of 980 AIMs across canine chromosome 10 (top), candidate gene *MSRB3* (middle), and candidate genes *PRKCE/EPAS1* (bottom). Three genetic groups are assumed for each analysis (K=2-4). Sample labels are provided on the X-axis.

**Supplemental Tables**

**Table S1.** Sample information.

| Sample ID | Species | Breed/population | Location | High/Low Land | Ancestry groups | Genome coverage | Supplemental Reference | NCBI SRA  Accession # |
| --- | --- | --- | --- | --- | --- | --- | --- | --- |
| DQ25 | Dog | Indigenous dog | Diqing, Yunnan, China | High (3300 m) | To test | ~15 | Gou et al. 2014 | SRP035294 |
| DQ28 | Dog | Indigenous dog | Diqing, Yunnan, China | High (3300 m) | To test | ~15 | Gou et al. 2014 | SRP035294 |
| DQ29 | Dog | Indigenous dog | Diqing, Yunnan, China | High (3300 m) | To test | ~15 | Gou et al. 2014 | SRP035294 |
| DQ32 | Dog | Indigenous dog | Diqing, Yunnan, China | High (3300 m) | To test | ~15 | Gou et al. 2014 | SRP035294 |
| DQ33 | Dog | Indigenous dog | Diqing, Yunnan, China | High (3300 m) | To test | ~15 | Gou et al. 2014 | SRP035294 |
| DQ45 | Dog | Indigenous dog | Diqing, Yunnan, China | High (3300 m) | To test | ~15 | Gou et al. 2014 | SRP035294 |
| DQ52 | Dog | Indigenous dog | Diqing, Yunnan, China | High (3300 m) | To test | ~15 | Gou et al. 2014 | SRP035294 |
| DQ54 | Dog | Indigenous dog | Diqing, Yunnan, China | High (3300 m) | To test | ~15 | Gou et al. 2014 | SRP035294 |
| DQ59 | Dog | Indigenous dog | Diqing, Yunnan, China | High (3300 m) | To test | ~15 | Gou et al. 2014 | SRP035294 |
| DQ60 | Dog | Indigenous dog | Diqing, Yunnan, China | High (3300 m) | To test | ~15 | Gou et al. 2014 | SRP035294 |
| DQZA01 | Dog | Tibetan Mastiff | Tibet/Qinghai/Yunnan, China | High (3300-5100 m) | To test | ~15 | Gou et al. 2014 | SRP035294 |
| DQZA06 | Dog | Tibetan Mastiff | Tibet/Qinghai/Yunnan, China | High (3300-5100 m) | To test | ~15 | Gou et al. 2014 | SRP035294 |
| DQZA12 | Dog | Tibetan Mastiff | Tibet/Qinghai/Yunnan, China | High (3300-5100 m) | To test | ~15 | Gou et al. 2014 | SRP035294 |
| DQZA18 | Dog | Tibetan Mastiff | Tibet/Qinghai/Yunnan, China | High (3300-5100 m) | To test | ~15 | Gou et al. 2014 | SRP035294 |
| DQZA19 | Dog | Tibetan Mastiff | Tibet/Qinghai/Yunnan, China | High (3300-5100 m) | To test | ~15 | Gou et al. 2014 | SRP035294 |
| DQZA23 | Dog | Tibetan Mastiff | Tibet/Qinghai/Yunnan, China | High (3300-5100 m) | To test | ~15 | Gou et al. 2014 | SRP035294 |
| DQZA33 | Dog | Tibetan Mastiff | Tibet/Qinghai/Yunnan, China | High (3300-5100 m) | To test | ~15 | Gou et al. 2014 | SRP035294 |
| DQZA55 | Dog | Tibetan Mastiff | Tibet/Qinghai/Yunnan, China | High (3300-5100 m) | To test | ~15 | Gou et al. 2014 | SRP035294 |
| DQZA80 | Dog | Tibetan Mastiff | Tibet/Qinghai/Yunnan, China | High (3300-5100 m) | To test | ~15 | Gou et al. 2014 | SRP035294 |
| DQZA81 | Dog | Tibetan Mastiff | Tibet/Qinghai/Yunnan, China | High (3300-5100 m) | To test | ~15 | Gou et al. 2014 | SRP035294 |
| dtm | Dog | Tibetan Mastiff | Lijiang, Yunnan, China | High | To test | 12.61 | Wang et al. 2013 | SRA068869 |
| bas | Dog | Basenji | AKC registered breed, USA |  | Reference | 12.6 | Freedman et al. 2014 | SRR2149861 |
| din | Dog | Dingo | Bargo Dingo Sanctuary, Australia |  | Reference | 25.75 | Freedman et al. 2014 | SRR2149867 |
| dc1 | Dog | Indigenous dog | Xi'an, China |  | Reference | 14 | Wang et al. 2013 | SRA068869 |
| dc2 | Dog | Indigenous dog | Simao, China |  | Reference | 15.2 | Wang et al. 2013 | SRA068869 |
| dc3 | Dog | Indigenous dog | Ya'an, China |  | Reference | 10.42 | Wang et al. 2013 | SRA068869 |
| dc4 | Dog | Indigenous dog | Gansu, China |  | Reference | ~17 | Wang et al. 2016 | SRA307300 |
| dc5 | Dog | Indigenous dog | Hebei, China |  | Reference | ~16 | Wang et al. 2016 | SRA307300 |
| dc6 | Dog | Indigenous dog | Shaanxi, China |  | Reference | ~17 | Wang et al. 2016 | SRA307300 |
| dc7 | Dog | Indigenous dog | Xinjiang, China |  | Reference | ~14 | Wang et al. 2016 | SRA307300 |
| dc8 | Dog | Indigenous dog | Shanxi, China |  | Reference | ~11 | Wang et al. 2016 | SRA307300 |
| dc9 | Dog | Indigenous dog | Guangdong, China |  | Reference | ~16 | Wang et al. 2016 | SRA307300 |
| dc10 | Dog | Indigenous dog | Guizhou, China |  | Reference | ~15 | Wang et al. 2016 | SRA307300 |
| dc11 | Dog | Indigenous dog | Yunnan, China |  | Reference | ~16 | Wang et al. 2016 | SRA307300 |
| dc12 | Dog | Indigenous dog | China/Vietnam border |  | Reference | ~17 | Wang et al. 2016 | SRA307300 |
| dc13 | Dog | Indigenous dog | China/Vietnam border |  | Reference | ~17 | Wang et al. 2016 | SRA307300 |
| inw | Wolf | Indian wolf | Koln Zoo | Low | Reference | 26.03 | Fan et al. 2016 | SRP044399 |
| irw | Wolf | Iranian wolf | Iran | Low | Reference | 27.94 | Fan et al. 2016 | SRP044399 |
| isw | Wolf | Israeli wolf | Neve Ativ, Golan Heights, Israel | Low | Reference | 21.6 | Freedman et al. 2014 | SRR2149870 |
| itb | Wolf | Italian wolf | Italy | Low | Reference | 13.01 | Zhang et al. 2014 | Distributed from author |
| crw | Wolf | Croatian wolf | Perković, Croatia | Low | Reference | 25.3 | Freedman et al. 2014 | SRR2149873 |
| ptw | Wolf | Portugal wolf | Portugal (N of Douro) | Low | Reference | 26.1 | Zhang et al. 2014 | Distributed from author |
| ru1 | Wolf | Central Russian wolf | Altai, Russia | Low | Reference | 12.38 | Wang et al. 2013 | SRA068869 |
| ru2 | Wolf | Eastern Russian wolf | Chukotka, Russia | Low | Reference | 9.05 | Wang et al. 2013 | SRA068869 |
| ru3 | Wolf | Western Russian wolf | Bryansk, Russia | Low | Reference | 13.17 | Wang et al. 2013 | SRA068869 |
| spw | Wolf | Spanish wolf | Spain | Low | Reference | 25.29 | Fan et al. 2016 | SRP044399 |
| LN1 | Wolf | Chinese wolf | Liaoning Province, North China | Low | Reference | 15.5 | Wang et al. 2016 | SRA307300 |
| SX1 | Wolf | Chinese wolf | Shanxi, China | Low | Reference | 12.6 | Wang et al. 2016 | SRA307300 |
| im1 | Wolf | Mongolian wolf | Inner Mongolia, China | Low | Reference | 24.6 | Zhang et al. 2014 | Distributed from author |
| im2 | Wolf | Mongolian wolf | Inner Mongolia, China | Low | Reference | 7.95 | Wang et al. 2013 | SRA068869 |
| im3 | Wolf | Mongolian wolf | Inner Mongolia, China | Low | Reference | 25.67 | Zhang et al. 2014 | Distributed from author |
| im4 | Wolf | Mongolian wolf | Inner Mongolia, China | Low | Reference | 22.93 | Zhang et al. 2014 | Distributed from author |
| im5 | Wolf | Mongolian wolf | Inner Mongolia, China | Low | Reference | 41.52 | This study | SRP096612 |
| xj1 | Wolf | Chinese wolf | Xinjiang, China | Low | Reference | 24.29 | Zhang et al. 2014 | Distributed from author |
| xj2 | Wolf | Chinese wolf | Xinjiang, China | Low | Reference | 26.87 | Zhang et al. 2014 | Distributed from author |
| xj3 | Wolf | Chinese wolf | Xinjiang, China | Low | Reference | 28.2 | Wang et al. 2016 | SRA307300 |
| xj4 | Wolf | Chinese wolf | Xinjiang, China | Low | Reference | 17 | Wang et al. 2016 | SRA307300 |
| xj5 | Wolf | Chinese wolf | Xinjiang, China | Low | Reference | 15.9 | Wang et al. 2016 | SRA307300 |
| qh1 | Wolf | Chinese wolf | Qinghai, China | High | Reference | 25.93 | Zhang et al. 2014 | Distributed from author |
| qh2 | Wolf | Chinese wolf | Qinghai, China | High | Reference | 26.44 | Zhang et al. 2014 | Distributed from author |
| qh4 | Wolf | Chinese wolf | Qinghai, China | High | Reference | 38.01 | Zhang et al. 2014 | Distributed from author |
| ti1 | Wolf | Chinese wolf | Tibet, China | High | Reference | 25.89 | Zhang et al. 2014 | Distributed from author |
| ti2 | Wolf | Chinese wolf | Tibet, China | High | Reference | 25.85 | Zhang et al. 2014 | Distributed from author |
| ti3 | Wolf | Chinese wolf | Tibet, China | High | Reference | 28.83 | This study | SRP096612 |

Fan Z, Silva P, Gronau I, Wang S, Armero AS, Schweizer RM, Ramirez O, Pollinger J, Galaverni M, Ortega Del-Vecchyo D, et al. 2016. Worldwide patterns of genomic variation and admixture in gray wolves. *Genome Res.* 26(2), 163-173. (doi: 10.111/gr.197517.115)

Freedman AH, Gronau I, Schweizer RM, Ortega-Del Vecchyo D, Han E, Silva PM, Galaverni M, Fan Z, Marx P, Lorente-Galdos B, et al. 2014. Genome sequencing highlights the dynamic early history of dogs. *PLoS Genet.* 10(1), e1004016. (doi: 10.1371/journal.pgen.1004631)

Gou X, Wang Z, Li N, Qiu F, Xu Z, Yan D, Yang S, Jia J, Kong X, Wei Z, et al. 2014. Whole-genome sequencing of six dog breeds from continuous altitudes reveals adaptation to high-altitude hypoxia. *Genome Res.* 24(8), 1308-1315. (doi: 10.1101/gr.171876.113)

Wang G-D, Zhai W, Yang HC, Fan R-X, Cao X, Zhong L, Wang L, Liu F, Wu H, Cheng L-G, et al. 2013 The genomics of selection in dogs and the parallel evolution between dogs and humans. *Nat. Commun.* 4,1860. (doi: 10.1038/ncomms2814)

Wang G-D, Zhai W, Yang H-C, Wang L, Zhong L, Liu Y-H, Fan R-X, Yin T-T, Zhu C-L, Poyarkov AD, et al. 2016. Out of southern East Asia: the natural history of domestic dogs across the world. *Cell Res.* 26(1), 21-33. (doi: 10.1038/cr.2015.147)

Zhang W, Fan Z, Han E, Hou R, Zhang L, Galaverni M, Huang J, Liu H, Silva P, Li P, et al. 2014. Hypoxia adaptations in the grey wolf (*Canis lupus chanco*) from Qinghai-Tibet Plateau. *PLoS Genet.* 10, e1004466. (doi: 10.1371/journal.pgen.1004466)

**Table S2.** Population membership proportions for 983 AIMs and for each ancestry outlier gene, *MSRB3* and *PRKCE*/*EPAS* in high elevation dogs.

See excel file: Supplemental_tableS2.xlsx
